# Assessing Consumer Willingness to Pay for Nutritional Information Using a Dietary App

**DOI:** 10.3390/nu14235023

**Published:** 2022-11-25

**Authors:** Seyyedehsara Sadrmousavigargari, Emilia Cubero Dudinskaya, Serena Mandolesi, Simona Naspetti, Seyed Mojtaba Mojaverian, Raffaele Zanoli

**Affiliations:** 1 Dipartimento di Scienze Agrarie, Alimentari e Ambientali (D3A), Università Politecnica delle Marche (UNIVPM), Via Brecce Bianche, 60131 Ancona, Italy; 2 Dipartimento di Scienze e Ingegneria della Materia, dell’Ambiente ed Urbanistica (SIMAU), Università Politecnica delle Marche (UNIVPM), Via Brecce Bianche, 60131 Ancona, Italy; 3 Department of Agricultural Engineering, University of Sari Agricultural Sciences and Natural Resources, 9th km of Farah Abad Road, Sari 4818168984, Iran

**Keywords:** choice experiment, food information, nutrition label, mixed multinomial logit, latent class

## Abstract

A healthy society is the foundation of development in every country, and one way to achieve a healthy society is to promote healthy nutrition. An unbalanced diet is one of the leading causes of noncommunicable diseases globally. If food was correctly selected and correctly consumed, both the problems of overeating and lack of nutrition could be largely solved while also decreasing public health costs. Interventions such as presenting necessary information and warning labels would help consumers make better food choices. Hence, providing nutritional information to consumers becomes essential. The present study investigates the importance of nutrition information labels on consumers’ preferences by estimating their willingness to pay for features and information provided by a dietary software program (app). An application can easily display the information to the consumers and help them make informed food choices. A discrete choice experiment investigated consumers’ preferences and willingness to pay to receive nutritional information. Mixed multinomial logit and latent class analysis were applied. The results showed the existence of heterogeneity in consumer preferences for different nutritional information provided by the application. Consumers are willing to pay more for salt and fat alerts. The results of this study allow for the analysis of consumers’ interest in nutritional information. Such results are essential for the industry for future investments in similar applications that potentially could help consumers make better informed choices.

## 1. Introduction

According to the World Health Organization [1], noncommunicable diseases (NCDs) such as cardiovascular diseases, cancers, chronic respiratory illnesses and diabetes are the leading cause of death worldwide. More than 41 million people die yearly from NCDs (71% of deaths worldwide), including 15 million individuals who die too early, between 30 and 69 years old. More than 85% of these early deaths in low- and middle-income countries are due to NCDs. NCDs are also considered a major health concern in developing countries like Iran. With a population of over 80 million, the mortality rate due to NCDs was almost 82% in 2016 [2]. In addition, in 2013, in Iran, a warning mortality growth of NCDs over the last 20 years was observed [3]. It is noteworthy that the ageing population of Iran can worsen the current situation [4].

Most early deaths are related to well-known risk factors, such as an unhealthy diet, harmful use of tobacco and alcohol, and lack of physical activity [1]. Dietary risk factors are the main contributors to NCDs [5]. People of all ages are more likely to experience an unhealthy diet than any of the other three factors (i.e., harmful use of tobacco, alcohol, and lack of physical activity). Foods rich in fat, saturated fats, trans-fats, sodium and sugar are likely to increase the risk of nutrition-related diseases [6]. Reducing such harmful elements in highly-populated countries such as Iran can be an effective strategy to control and manage NCDs [7]. Therefore, steps must be taken to guide appropriate food consumption behaviours [4]. If foods were accurately selected and consumed, the problems of overeating and lack of nutritional value could be solved, to a large extent, and could help decrease public health costs [8].

Numerous factors affect consumers’ food choices [9]; among them is the information displayed on food labels. As an essential tool to inform consumers, food labelling is the primary means of exchanging information between producers and consumers along the food supply chain [10]. Consumers look for different types of food information related to religious constraints, the presence of allergens, environmental issues and production procedures [11]. In this vein, nutritional food labelling has gained importance in the development of food industries with increasing competition and consumers’ greater attention to health factors [10]. Food nutritional labels influence consumers’ buying decisions and change their behaviour toward a healthy and desirable food pattern [12,13]. Therefore, presenting nutritional and allergen information on the food label is considered part of a broad attempt to prevent the health cost of food-related diseases [14].

Although the information on the food package is simple, consumers seem to use such information less than one might expect [8]. Because of limited attention, customers pay attention to a restricted number of product features. Usually, only those that may be relevant as a quality cue are considered: price and brand are the most relevant [15]. During their in-store experience, consumers are also influenced by various marketing stimuli, and since they intend to shop a wide range of products, they face multidimensional decision-making problems.

Time pressure and cognitive limitations are significant constraints in using and understanding label information in real shopping scenarios. Consumers are not willing to consider the health labels, especially when constrained by time [15]. Grunert et al. [16] concluded that only 27% of the consumers check the nutritional information of the food packages. Moreover, although the majority (83%) of Iranian consumers declared to read food labels when shopping, only a small percentage of them (5%) aimed to obtain nutritional information on labels [8,17]. A possible reason is that the consumers are too hurried to look at and analyse nutritional and health-related data presented on food labels due to a distracting and crowded purchasing atmosphere. In this situation, evaluating the nutritional value of their shopping basket could be difficult even for those conscious consumers who wish to choose healthy foodstuffs [14].

Using interpretation guides, such as software programs (or applications–apps) and keeping a tally of nutritional information on food products while shopping could help consumers to make more conscious choices [12,14,18]. This study investigates consumers’ preferences and willingness to pay for different dietary software program information features. A discrete choice experiment was applied to evaluate the hypothetical services mentioned. Thus, three models were estimated: a Mixed Multinomial Logit (ML) in preference space, a ML in willingness to pay (WTP) space, and a latent class. The findings of this study are useful to several stakeholders. Policymakers could use the present results to develop policies and favour software applications that allow more conscious food choices and promote a healthy change in people’s diet. Consumers could also benefit from having access to a tool that assists them in selecting their food.

## 2. Literature Review

According to the literature, food labelling, by providing relevant information to consumers, has a significant role in healthy food choices [19]. Front Of Package (FOP) nutrition labels are food labels that provide nutritional information on the most common intakes of saturated fat, sugar, and sodium (or salt) in various designs and assist consumers in making healthy food choices [20,21]. Emrich et al. [22] found that consumers who consulted FOP nutrition labels reduced unhealthy food in their diets, such as overall total fat, saturated fat, and sodium intake. Similarly, Mclean et al. [23] showed that people with hypertension would lower high-sodium processed food intake using FOP nutrition labels.

Consumers consider nutrition labels essential in evaluating the product [24]. However, the information included in the labels does not influence their purchasing decisions. Barreiro-Hurlé et al. [25] showed that using nutritional information, regardless of the type of the nutritional label (facts panels or claims), leads consumers to a healthy food selection.

In addition, background knowledge is needed to understand the information on the nutritional labels [26,27]. Low awareness and lack of nutritional knowledge are the most important reasons for not paying attention to the nutritional contents of food labels [17]. Therefore, to be effective, salient and personalised information should be easily, clearly, and quickly accessed through reliable tools that lead to informed food choices [28].

A possible efficient solution to all the mentioned issues is using technology; usability is of paramount relevance here [29]. In other words, smartphone applications can be used to read and process the written information on nutritional labels and display the information to consumers in an easy-to-understand way. Moreover, by adapting to consumers’ unique features and providing nutritional advice, these apps can assist in choosing the best product that consumers require [30]. In this regard, apps such as “Tapingo” or “SmartAPPetite” were developed to provide consumers with relevant information worldwide and in southwestern Ontario. Whereas university students used the first app to order food, the second was used to motivate people to consume local food, considering their nutritional preferences and providing customised information [31,32]. Neither of the apps are available in Iran [31,32]. Therefore, customised food information, specific for each individual, easily understood and relevant to their needs, can be provided via technological tools such as the apps mentioned above. Moreover, previous studies have found that participants were willing to pay for customised food information [14]. However, the willingness to pay was mainly for the information that included allergy-alert and diet-alert warnings.

This information could also be sold based on the subscription method. Several economic factors explain the existence of subscriptions. First, subscriptions can reduce transaction costs. In other words, several products can be exchanged only once and not every time the product is supplied and used. Second, the risk of price change is less for the consumer, although the seller may encounter uncertainty about future prices. However, the payment in advance is a premium for running that risk. Finally, subscriptions can lower market uncertainty by fixing the number of products sold. Subscriptions allow sellers to segment buyers into groups with demand elasticities and thus permit price discrimination, which can benefit both producers and social welfare, so long as the marginal cost of production is positive. There are no binding capacity constraints [33].

Another critical issue in using the app is the information provision format. Previous studies presented and analysed two main label formats: the Guideline Daily Amount (GDA) and Traffic Light System (TLS). The GDA shows nutritional information numerically and positively influences healthy food choices [34]. The TLS is widely used in the food industry. It typically shows different lights (e.g., green, amber or red labels) to inform whether foods contain unhealthy ingredients (e.g., low, medium or high amounts of salt, fat, saturated fat or sugars) [35]. According to several researchers [36,37], this system also has a significant role in the selection of healthy food.

However, the information provision format plays a crucial role in forming the consumers’ perspective toward the nutritional information provision. Consumers like nutritional labels with nice colours, symbols, and easy-to-understand information, whether graphically or numerically [18]. In a study including TLS and GDA, approximately 90% of participants checked to agree/strongly agree on the scale when asked if they liked TLS [38]. In the same study, GDA was liked by only 50% of the participants. Similarly, consumers in New Zealand preferred the TLS format most often [39]. By contrast, other studies found that GDA was considered a more attractive and liked label than TLS [40,41]. Different factors such as social level, local differences, interest in healthy eating and nutritional knowledge of consumers play a significant role in utilising GDA and understanding nutritional information [27]. Young consumers with no children using GDA are more interested in nutritional information and more aware of food-related health issues [12].

Another factor influencing consumers informed food choice is the difference between displaying food information for every single product in the shopping basket or the total number of items in an aggregate basket. The limited number of studies on this issue have found that consumers prefer to process each product’s information individually rather than obtain aggregate information for the total basket [12,14]. However, given the scarcity of literature on this issue, further research is needed.

## 3. Materials and Methods

### 3.1. The Discrete Choice Model

A Discrete Choice Experiment (DCE) is a survey-based methodology widely used to model consumers’ preferences [42]. According to the method, all goods and services are defined by a set of attributes. Therefore, respondents are presented with several market simulations (choice sets) in which the offered products or services differ in at least one attribute [43]. All alternatives shown to the respondents are evaluated using the same list of attributes but with different levels (e.g., the presence or not of the organic logo). Based on these attributes and their levels, participants are asked to choose the alternative they preferred the most in each choice set, according to the characteristics and price of the presented products or services. Including the price/cost of each product enables the calculation of a respondent’s willingness to pay (WTP) for a specific attribute.

The DCE is based on the Lancastrian consumer theory [44] and the Random Utility model (RUM) [45]. According to Lancaster [44], individuals’ choice is based on the utility maximisation rule, meaning they will select the alternative that gives the highest utility [46]. Moreover, the RUM framework indicates that the utilities of different goods or services can be broken down into separate utilities for their attributes. Therefore, the total utility of the selected item *i* by the individual *n* is represented as the sum of two utility components, a systematic component ($V_{ni}$) and a non-observed component ($\varepsilon_{ni}$), which is treated as random. The systematic (non-observed) component can be further approximated by a linear function of the product or service attributes in the vector $X_{ni}$, while the population utility weights for each attribute can be collected through the vector β:

(1) $$U_{ni}=V_{ni}+\varepsilon_{ni}=\beta X_{ni}+\varepsilon_{ni}$$

Under the assumption that $\varepsilon_{ni}$ follows an independent and identically extreme value distribution, a multinomial logistic (MNL) model can be implemented. However, the main limitation of the MNL model is that it assumes homogeneity of preferences across consumers, which is an unrealistic assumption [47]. Flexible models such as the Mixed Multinomial Logit (ML) can capture unobserved preference heterogeneity across individuals. Assuming that the unknown parameter estimating β is random according to the continuous probability distributions, the utility of the individual *n* from alternative *i* is specified as:

(2) $$U_{ni}=\beta^{\prime }X_{ni}+\varepsilon_{ni}$$

where β′ varies between individuals but not over alternatives (representing ‘consumers’ preferences heterogeneity); while $X_{ni}$ is a vector of observed variables related to the alternative *i* and decision-maker n. *ε_ij_* is a random term distributed i.i.d. extreme value over individuals and alternatives.

In the present study, the random parameters β were assumed to be normally distributed to allow positive and negative preferences for each attribute. Only the price parameter was assumed to be distributed following a negative log-normal distribution to obtain a better fit with the microeconomic theory (negative utility for the price parameter).

Furthermore, due to the impossibility of direct interpretation of coefficients in the preference space, the willingness to pay was also calculated. As the ML models accounts for the heterogeneity of preferences, the WTP was directly estimated in the WTP-space which allows to interpret coefficients of attributes and compare them with each other in and easier way, while providing more reasonable distributions [48]:

(3) $$U_{ni}=\alpha_{ni}-\lambda_{n}p_{ni}+{\left(\lambda_{n}\gamma_{n}\right)}^{\prime }\;X_{ni}+\varepsilon_{ni}$$

where $\lambda_{n}=\left(\beta_{n\;price}/\mu_{n}\right)$ with $\beta_{n\;price}$ being an individual-specific coefficient for the price, while *μ_n_* represents an individual-specific scale parameter $\gamma_{n}=\left(c_{n}/\lambda_{n}\right)$, where $c_{n}=\left(\beta_{n}/\mu_{n}\right)$.

Although the ML models consider variety in preferences, the source of the heterogeneity of tastes is unknown. Moreover, their specification requires an a priori assumption for the β distribution. These limitations can be overcome with the latent class (LC) models [49,50], providing a variety of information about participants’ behaviour. In this model, the heterogeneity of preferences is accommodated by dividing consumers into a set of exclusive classes with homogeneous preferences within them. Therefore, the utility in the LC models of the individual n for the alternative *i* is:

(4) $$U_{ni|c}=\beta_{c}X_{ni}+\varepsilon_{ni|c}$$

where β*_c_* is the vector of class *c* associated with a segment-specific vector of coefficients, while *ε_ni|c_* follows a Gumbel distribution.

The class membership of the individuals is a priori unknown to the analyst, as it depends on the observable attributes and the latent unobservable components [51]. The optimal number of classes was identified using the Akaike Information Criterion (AIC) and Bayesian Information Criterion (BIC), as well as the significance of the estimated parameters and the interpretation of the model (in terms of sign and size of the parameters) [49,52]. Based on the assumption of linearity of the utility function, the indirect utility for each alternative *i* for each segment *c* is:

*V_i|c_* = *α_i|c_* + *β*_Basket_*X*_Basket,*i|c*_ + *β*_Format_*X*_Format,*i|c*_ + *β*_Salt alert_*X*_Salt alert,*i|c*_ + *β*_Fat alert_*X*_Fat alert,*i|c*_ + *β*_Allergy alert_*X*_Allergy alert,*i|c*_ + *β*_payment_
*X*_payment,*i|c*_ + *β*_Price_*X*_Price,*i|c*_ + *ε_i|c_* (5)

The WTP for each attribute was estimated for each segment. The WTP was calculated as the price change related to a unit increment un a specific attribute in that segment:

(6) $$W\mathrm{TP}=-\frac{\beta_{\mathrm{attribute}}}{\beta_{\mathrm{price}}}$$

where $\beta_{\mathrm{attribute}}$ is the coefficient of the attribute of interest in a specific segment and $\beta_{\mathrm{price}}$ is the price coefficient in the same segment.

### 3.2. Product and Attribute Selection

A new digital mobile application that helps consumers make informed food choices during grocery shopping was selected for the analysis. It can provide consumers with personalised information on potential unhealthy components (e.g., fats, salt) in the food products. A warning on possible allergies is also available. This information can be provided individually or as a basket for all products simultaneously. Moreover, the information can be displayed using a TLS or GDA. The service has a price in local currency, which varies according to the affiliation program (monthly, quarterly or yearly). Hence, seven attributes were selected based on previous literature: basket [12,14,53], format [24,34,54,55], fat alert [6,19,22,56,57], salt alert [23], allergy alert [58,59,60] payment type [61,62,63], and price [12,14]. The description of attributes and their levels is presented in Table 1.

### 3.3. Data Collection and Analysis

The data were gathered through personal interviews between August and September 2019 in Sari, the capital of Mazandaran (Iran). A convenient sample in the province of Mazandaran was selected given the predominance of nutritional-related diseases. In Mazandaran, the leading cause of death is cardiovascular disease. Moreover, Mazandaran presents the higher levels of obesity prevalence in all of Iran and is among the top five provinces regarding the prevalence of hyperlipidaemia and hypertension [64].

The individuals included in the study were over 18 years old, currently living in Sari and had an income or could use the household income for food expenses. For the sample collection, three leading chain supermarkets were selected during quiet hours of the department store (excluding Thursdays and Fridays), so the respondents would not get distracted. Customers and staff present at the moment of the study were randomly recruited. Out of the 207 distributed questionnaires, three incomplete questionnaires were excluded. The final sample included 204 questionnaires.

The questionnaire was developed in English on the Qualtrics platform. Then, it was translated and back-translated to Farsi [65]. Close collaboration with a local researcher allowed conceptual, functional and categorical equivalence in the translation [66]. The questionnaire included socio-demographic data, health conditions, shopping behaviour, awareness of food-related diseases and the DCE.

### 3.4. Discrete Choice Experiment Design and Estimation

Given the seven attributes and their levels, 384 (25 × 3 × 4) possible alternatives could be created. The number of combinations was reduced to obtain a more reliable and statistically efficient design. A fractional factorial design of 12 unlabelled choice sets was developed employing a D-efficient design in the Ngene software (D-error = 0.35 and A-error = 0.40). Each respondent was presented with 12 choice sets with two alternatives and a “no choice” option in case the respondents would prefer not to choose either the application services. All choice sets and alternatives were presented in a randomised order to avoid any bias [67].

**Figure 1:**
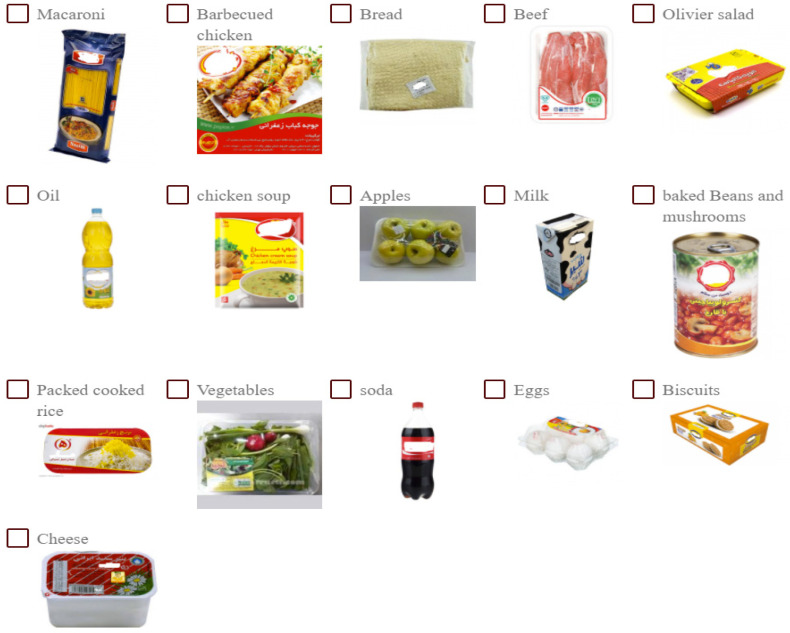
List of products showed to the respondents.

Before the choice experiment, participants were introduced to the purpose of the research. Then, the respondents were presented with a list of products, in their local language, based on the criteria of “optimal food basket for Iranian households” introduced by the Ministry of Health, Treatment and Medical Training of Iran [68]. The list was developed based on the consumers’ nutrient requirements according to age and gender, the cost (including only economically affordable goods) and the production potential in the country for each product. Both raw and pre-prepared products were included. The products were presented in a graphical format (Figure 1) and randomised order.

Individuals were asked to imagine they were in a store and choose the groceries they usually buy during their shopping trip to simulate purchasing decision-making. The participants could choose as many products as they wanted. The weight/volume of each product was specified (Table 2). The objective of this task was to get the consumer to visualise the products they usually buy and evaluate how useful the proposed application could be when valuing the nutritional properties of their usual food shopping.

**Figure 2:**
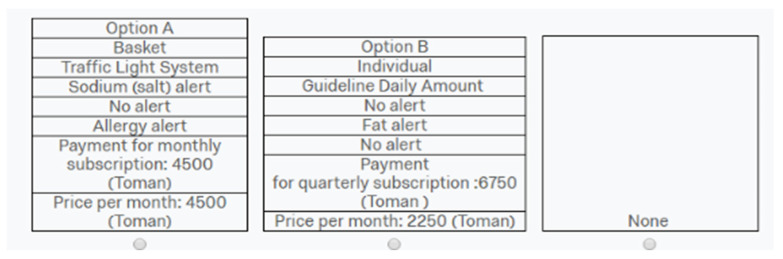
Example of choice set.

Then, the respondents were introduced to the diet application. The participants were told that a new diet application was being launched to aid customers in making healthier food choices. The service would allow them to keep a tally of the main nutritional components of the food they purchase, as well as the presence of specific harmful substances for them (e.g., sugar, fat or allergic components). Therefore, the application would display in an easy-to-read format the nutritional information by scanning the bar code of the selected product. All attributes of the application and the attributes’ levels were presented and thoroughly described to inform participants equally. Participants could also request help or ask for clarifications to the interviewer present at the moment of the development of the study. An example of a choice set (Figure 2) was also introduced to the participants, followed by a “cheap talk” to reduce the hypothetical bias [69].

In the DCE, participants were asked to select the alternative with the features they would like the most for the healthy application at the given price and payment method. All alternatives were simultaneously presented as in the example in Figure 2. Respondents had no time limit for their selection. A total of 2448 choices were collected.

The data were analysed through the APOLLO package in R [70]. An MNL model was estimated as a departure point, followed by a ML in the preference-space using Halton draws with 500 replications. Coefficient estimates from the ML in the preference space were incorporated as priors for the ML estimations in the WTP-space, using Halton draws with 500 replications. To facilitate convergence in the WTP-space, a scaling factor was implemented.

## 4. Results

The characteristics of the sample are shown in Table 3. A total of 204 responses were collected. The average age of the participants was 37 years old. In addition, 36.27% of respondents declared they are the head of their household, meaning they were responsible for providing all or most of the household expenses or deciding how to spend the household income. Most respondents presented a low income, and the average household size was about 3.5 people. The average body mass index (BMI) was 27 (kg/m^2^), indicating that people in the sample suffered from being overweight [71].

Regarding the results of the ML Table 4, the alternative specific constant (ASC) was positive and statistically significant. That is, choosing alternatives A or B yielded positive utility. The basket coefficient was negative and statistically significant. This means that the respondents preferred to have displayed the product information individually rather than in an aggregated format for all items. The format coefficient was not statistically significant, indicating the participants’ indifference to format. Presenting information using the Traffic Light System (TLS) or Guideline Daily Amount (GDA) did not make any difference to the respondents.

Additionally, the coefficients of salt alert, fat alert, and allergy alert were positive and statistically significant, indicating that consumers in Sari would like to know whether their chosen products have a high intake of fat and salt in compared to not having such information (reference level). They also preferred to be informed about the products that could cause them food allergies compared to not having such information

Regarding payment frequency, respondents prefer a quarterly payment over a monthly payment (reference level). However, a monthly payment was preferred over a yearly one. The price coefficient was significant and negative, which means that participants preferred to pay less. Dispersion parameters (standard deviation estimates) were statistically significant, exhibiting heterogeneous preferences for all attributes.

Based on the Mixed Multinomial Logit (ML) results in the willingness to pay space in Table 4, the highest willingness to pay was related to the fat alert. Consumers were prepared to pay approximately 23,000 tomans (approximately €4.93) to receive fat alerts for selected products. Following, consumers were willing to pay about 21,000 tomans (approximately €4.50) to receive the salt alerts and 13,000 tomans (approximately €2.79) to receive allergy alerts.

Most of the standard deviation estimates for the WTP model were significant, indicating a great variety among consumers’ preferences for these attributes. However, the standard deviation estimate for the format coefficient was not significant, meaning that there was no heterogeneity among respondents regarding their willingness to pay for this attribute. In general, respondents are not willing to pay to get their nutritional information in the TLS format over the GDA, and this is quite a homogeneous result across the sample.

A latent class model was estimated to investigate the source of such heterogeneity in consumer behaviour. The number of classes was based on the model with the lower BIC (3281.51) and the best interpretability. Thus, participants were divided into two groups with different behavioural characteristics. Accordingly, 14% of consumers belonged to class one and 86% to class two. The result of the model is reported in Table 5.

Results of the latent class model showed significant differences between the two classes. The respondents in class 1 were indifferent to receiving the information for each product or as a basked. Moreover, the format was negative and statistically significant, demonstrating consumers’ preference for GDA rather than TLS. The coefficients of the salt and fat alerts were positive and statistically significant, indicating the preferences of the consumers to receive the alerts related to the prevention of health issues. The allergy alert estimate was only significant at 10%. In this class, the quarterly and annual payments coefficients were negative and statistically significant, indicating the respondents’ preferences for a monthly subscription over a quarterly and annual subscription. The coefficient of the price was negative as expected and statistically significant.

In contrast to class 1, in class 2, the basket attribute was significant, which indicated that people in this class preferred that the diet app presents nutritional information for each product separately rather than for the entire basket. The influence of format attributes on consumer preference was not statistically significant in class 2. Moreover, salt alert, fat alert, and allergies alert were positive and statistically significant. Unlike in class 1, the payment attributes were not statistically significant. Participants were indifferent to monthly, quarterly, and annual subscriptions. The coefficient of the price in this class was also negative, as expected.

In summary, although the salt alert and fat alert coefficients were higher in class 1 compared with class 2, the willingness to pay for the salt alert and fat alert in class 2 was larger than in class 1. Similarly, the willingness to pay for the allergy alert was higher in class 2 than in class 1. While the consumers in class 1 had clear preferences for the format (GDA) and the payment frequency (monthly), consumers in class 2 were indifferent to these attributes. On the other hand, participants in class 2 preferred to receive information for each product, while participants in class 1 did not have any specific preference for it. In addition, the ASC was positive and significant, indicating positive preferences for using the application.

## 5. Discussion

The present study uses a discrete choice experiment to investigate consumers’ preferences and WTP for receiving nutritional information provided by a dietary application during grocery shopping. The findings from the ML model indicate heterogeneity in consumer preferences for the different attributes of the app. The aggregated results from the ML and the latent class models showed that participants were willing to pay for customised information at the point of the purchase. This result is consistent with previous literature [12,14], which showed that consumers preferred obtaining dietary and allergy information when buying food. This result also aligns with previous findings that about 80% of Mexican consumers liked and wanted warning labels on the front of food packages [38].

Moreover, considering both models’ positivity and the significance of salt and fat alert coefficients, respondents are willing to pay for information that helps them make informed and healthier food choices. In this regard, it is recommended that the food industry in Iran insert alerts (e.g., salt alert and fat alert) on food packaging, similar to the action taken by the tobacco industry.

In addition, the ML and latent class models results inferred that most participants preferred to receive the customised information from the application for each product individually, rather than for the whole basket as aggregated information. In other words, individuals, similarly to previous findings [14], prefer to examine the nutritional information for each product individually. However, in the latent class, 14% of individuals were indifferent to obtaining information in this context. This result is also in line with previous research [12], which found that 89% of consumers were indifferent whether the information was provided product by product or as an aggregated format for the whole basket. A possible explanation for this can be the lack of such devices in Iran to display integrated information of the shopping basket at the purchasing time; therefore, this feature is intangible to some individuals.

The result of the ML indicated that consumers were indifferent about the format of the nutritional information. However, the latent class analysis showed that the smallest of the resulting classes (14%) preferred nutritional information displayed in Guideline Daily Amount rather than the Traffic Light System. Although this finding is in line with previous research [40], the results are somewhat surprising, given that the TLS has been mandatory in Iran since 2016, and the GDA is optional [10]. A possible reason is that consumers are still not familiar with TLS. A study showed that 59% of the participants were not familiar with the Traffic Light System and only 27% of consumers claimed that they had used the TLS [17]. It is worth mentioning that before the implementation of TLS, the nutritional information was presented on the food packaging, similar to the GDA format. According to previous research, although the GDA format presents more detail [72] and its preference might be influenced by the level of education [73], it is also liked more by consumers than other formats [74]. Therefore, designing educational programs in health centres to introduce labels and their function in informed food choice should be a priority for responsible organisations.

The results of the ML also indicated the existence of a strong preference for monthly payments rather than yearly. Consumers are unwilling to undertake a long-time commitment [75]. Moreover, the significant standard deviation estimates for the payment methods in the ML model show high heterogeneity among respondents. So, although a class of respondents prefer monthly payments (class 1), the other class is indifferent. In the case of implementing the app, various payment frequency alternatives should be presented to address different segment needs.

In general, the results of the present paper contribute to the literature in two ways. First, to the authors’ best knowledge, there are no previous studies on the WTP of Iranian consumers for features of an app that could help them make a more informed decision [12,14,18]. Second, given that the geographical area in which the sample was collected there is an important predominance of nutritional-related diseases [64], it is key to identify communication strategies and tools to inform consumers and assist them in their food choices effectively. The present study explores one possible alternative among many others. However, future research should also compare this option with other digital and non-digital solutions.

## 6. Conclusions

In this study, a DCE was developed and analysed to examine the effect of a diet app, which included the characteristics of the basket, format, salt alert, fat alert, allergy alert, payment, and price on consumers’ preferences and their willingness to pay in Sari, Iran.

Our findings provide new insights into consumers’ preferences for the nutritional information provided by the application as an effective tool in helping consumers to make conscious choices during their shopping experience. In this regard, policymakers could benefit from our results by encouraging the implementation of diverse systems (digital and non-digital) that assist consumers in making an informed decision during their grocery shopping. Meanwhile, future research should also explore how to effectively deliver this information to the consumers, studying the diverse tools that could be used for this purpose (e.g., apps, advertisement, etc.), how to frame the messages effectively, as well as consumer acceptability.

Policymakers should also promote campaigns that increase consumers knowledge regarding the presence or lack of nutrients in certain foods. This could collaborate in reducing the incidence of non-communicable diseases by encouraging more conscious food choices. Such a process should also motivate the food industry to increase sales by changing food formulation to low-salt and low-fat products

The present study also presents some limitations. First, the study includes a convenient sample of 204 people living in Sari, the capital of Mazandaran (Iran). Although the geographical area of the study was chosen on purpose given the predominance of nutritional-related diseases in the area [64], the results are not generalisable to the rest of the Iranian population or other countries. Future research would benefit in replicating the current study in other geographical areas of Iran or other countries with similar morbidity profiles.

Second, the present results are based on a hypothetical market, which means that the hypothetical bias may arise [76]. Respondents might express certain preferences in the hypothetical DCE that may differ from their actual preferences under real circumstances [77], leading to overestimated coefficients [78]. Therefore, future work should benefit greatly by combining revealed and stated preference data.

## Figures and Tables

**Table 1:** Features of the hypothetical dietary application.

Attribute	Description	Levels *
Basket	The nutritional information is displayed in two ways: a: basket: information shown in aggregate form for all the selected products (the entire basket). b: individual: information presented for each product in the basket.	Basket = 1, *Individual = 0*
Format	a: Guideline Daily Amounts (GDAs): this label communicates nutrient content levels in absolute values, per 100 g or portion size, and is also expressed as a percentage of proposed daily reference quantities within one’s total diet. b: Traffic Light System (TLS): this label shows nutrient content by weight. Green, amber, and red colours are used to respectively depict low, medium, and high content for unhealthy components (e.g., salt).	*Guideline Daily Amounts = 0*, Traffic Light System = 1
Sodium (salt) alert	A service will alert shoppers if they buy products with high sodium content.	Salt alert = 1, *No alert = 0*
Fat alert	A service to alert shoppers to avoid buying foods with high-fat content.	Fat alert = 1, *No alert = 0*
Allergies	A service helps buyers avoid an allergic reaction. The most common ingredients that trigger food allergens include dairy, eggs, peanuts, wheat, corn and soy. The service also includes any allergens found in flavourings, colourings or other additives. This application allows users to scan products’ barcodes to check for allergens.	Allergy alerts = 1, *No alerts = 0*
Payment	Payment for using the diet application is based on monthly, quarterly, or yearly subscription. Quarterly and yearly subscribes have access to a 50% and 70%discount, respectively.	*Monthly = 0*, Quarterly = 1, Yearly = 2
Price per month	Monthly cost for using services of diet application (without the quarterly and yearly discount).	1500-, 4500-, 7500-, 10,500-(tomans)

* The reference level for categorical attributes is presented in *italics*.

**Table 2:** List of products presented to participants and their weight/volume.

List of Products	
Bread (400 g package)	Barbecued chicken (800 g pack)
Rice (300 g pack)	Eggs
Macaroni (500 g package)	Milk (1 L)
A can of baked beans and mushrooms (400 g)	Vegetable oils (810 g)
Olivier salad (250 g pack)	Chicken soup (70 g package)
Vegetables (250 g pack)	Biscuits (100 g)
Apples	Coca-cola (1.50 L)
Cheese (450 g)	Please add to list other foodstuffs you buy

**Table 3:** Sample characteristics.

Variable	Percent (%)
*Gender*	
Female	57.00
Male	43.00
*Marital Status*	
Single	29.41
Married	70.59
*Age*	
18–30	25.98
31–50	61.27
51–65	12.25
Over 65	0.49
*Education level*	
Primary (1–5)	2.45
Middle (6–8)	6.86
Diploma	20.59
Associate degree	13.24
Bachelor	32.35
Master or over	24.51
*BodyMass Index (BMI)*	
Underweight	3.90
Normal Weight	41.00
Pre-obesity	40.00
Obesity	16.00
*Health conditions*	
High blood pressure	15.00
Food allergies	15.00
*Average household monthly income (10 million rial)*	
Below 1.49	19.61
1.50–2.49	29.90
2.50–3.49	21.57
3.50–4.49	11.76
4.50–5.49	7.35
5.50–6.49	3.43
over 6.50	6.37

**Table 4:** Results of the ML in preference space and in WTP-space.

	ML in Preference Space		ML in Willingness to Pay Space (1000 Tomans)	
Variable	Mean	Standard Error	Mean	Standard Error
ASC	1.94 ***	0.20	−1.67	−0.20
*Mean estimates*				
Basket	−0.47 ***	0.09	−9.01 *	5.09
Format (TLS)	0.03	0.07	−0.19	1.14
Sodium (salt) alert	1.16 ***	0.10	+20.70 ***	7.10
Fat alert	1.33 ***	0.10	+23.04 ***	7.95
Allergy alerts	0.79 ***	0.09	+12.92 ***	4.02
Payment quarterly	1.56 *	0.80	+23.06	15.17
Payment yearly	−0.22 **	0.11	−1.74	2.00
Price	−2.64 ***	0.24	+2.71 ***	0.32
*Standard deviation estimates*				
Basket	0.96 ***	0.10	16.47 ***	5.47
Format	0.28 **	0.14	3.36	2.10
Sodium (salt) alert	0.78 ***	0.09	11.98 ***	4.09
Fat alert	0.90 ***	0.11	10.65 ***	3.18
Allergies	0.74 ***	0.10	12.05 **	5.73
Payment quarterly	2.22 ***	0.54	30.64 **	12.40
Payment yearly	0.47 ***	(0.15)	6.79 **	3.29
Price	0.76 ***	(0.13)	0.89 ***	0.20
Estimated parameters	17		17	
Log-likelihood (final)	−1504.87		−1496.26	
Rho-square	0.44		0.44	
Adj.Rho-square	0.43		0.44	
AIC	3043.75		3026.52	
BIC	3142.4		3125.17	

Notes: symbols *, **, *** mean significant at 10%, 5% and 1%. ASC captures the choice of either alternative A or B as opposed to the “none” option. All estimates follow a normal distribution, besides price (negative log-normal).

**Table 5:** Results of the MNL latent class model.

	Class 1				Class 2	
Variables	**Mean**	**Standard Error**	WTP (1000 Tomans)	**Mean**	**Standard Error**	WTP (1000 Tomans)
Basket	−0.54	0.37		−0.36 ***	0.06	−7.2
Format	−1.13 **	0.49	−2.51	0.04	0.05	
Sodium (salt) alert	1.96 ***	0.70	4.36	0.81 ***	0.05	16.2
Fat alert	3.98 ***	0.79	8.84	0.71 ***	0.06	14.2
Allergy alerts	0.78 *	0.41	1.73	0.58 ***	0.05	11.6
Payment quarterly	−2.88 ***	0.75	−6.40	−0.02	0.32	
Payment yearly	−2.49 ***	0.73	−5.53	−0.07	0.09	
Price	−0.45 ***	0.11		−0.05 ***	0.01	
ASC ^a^	2.12 ***	0.19		-		
Class membership probability	0.14			0.86		
Log-likelihood (final)	−1570.53					
Rho-square	0.42					
Adj.Rho-square	0.41					
AIC	3177.06					
BIC	3281.51					

Notes: symbols *, **, *** mean significant at 10%, 5% and 1%. ^a^ ASC captures the choice of either alternative A or B as opposed to the “none” option.

## Data Availability

Data are available from authors upon request.
